# Kounis Syndrome—not a Single-organ Arterial Disorder but a Multisystem and Multidisciplinary Disease

**DOI:** 10.4274/balkanmedj.galenos.2019.2019.5.62

**Published:** 2019-07-11

**Authors:** Nicholas G. Kounis, Ioanna Koniari, Dimitrios Velissaris, George Tzanis, George Hahalis

**Affiliations:** 1 Department of Cardiology, Patras University School of Medicine, Patras, Greece; 2 Electrophysiology and Device Department University Hospital of South Manchester NHS Foundation Trust, Manchester, UK; 3 Department of Internal Medicine, Patras University School of Medicine, Patras, Greece; 4 Interventional Cardiology Unit, GVM Care & Research Maria Cecilia Hospital, Via Madonna di Genova, Cotignola RA, Italy

**Keywords:** Allergy, anaphylaxis, hypersensitivity, Kounis syndrome

## Abstract

Coronary symptoms associated with conditions related to mast cell activation and inflammatory cell interactions, such as those involving T-lymphocytes and macrophages, further inducing allergic, hypersensitivity, anaphylactic, or anaphylactic insults, are currently referred to as the Kounis syndrome. Kounis syndrome is caused by inflammatory mediators released during allergic insults, post-inflammatory cell activation, and interactions via multidirectional stimuli. A platelet subset of 20% with high- and low-affinity IgE surface receptors is also involved in this process. Kounis syndrome is not just a single-organ but also a complex multisystem and multi-organ arterial clinical condition; it affects the coronary, mesenteric, and cerebral arteries and is accompanied by allergy–hypersensitivity–anaphylaxis involving the skin, respiratory, and vascular systems in the context of anesthesia, surgery, radiology, oncology, or even dental and psychiatric medicine; further, it has significantly influences both morbidity and mortality. Kounis syndrome might be caused by numerous and continuously increasing causes, with broad clinical symptoms and signs, via multi-organ arterial system involvement, in patients of any age, thereby demonstrating predominant anaphylactic features in terms of a wide spectrum of mast cell-association disorders. Cardiac symptoms, such as chest pain, coronary vasospasm, angina pectoris, myocardial infarction, stent thrombosis, acute cardiac failure, and sudden cardiac death associated with subclinical, clinical, acute, or chronic allergic reactions, constitute the clinical manifestations of this syndrome. Since its first description, a common pathway between allergic and non-allergic coronary events has been demonstrated. The hypothesis is based on the existence of a much higher degree of mast cell degranulation at plaque erosion or rupture sites compared with at the adjacent areas or even more distant segments in post-acute myocardial infarction of non-allergic etiology. Although mast cell activation, differentiation, and mediator release takes days or weeks, the mast cell degranulation may occur just before any acute coronary event, further resulting in coronary artery vasoconstriction and atheromatous plaque rupture. It seems that medications and natural molecules stabilizing the mast cell membrane as well as monoclonal antibodies protecting the mast cell surface can emerge as novel therapeutic modalities for acute coronary and cerebrovascular event prevention.

Allergic, hypersensitivity, anaphylactic, or anaphylactoid reactions associated with cardiovascular symptoms and signs appeared more than 7 decades ago as per the English, German, and Austrian medical literature (1,2). Such reactions were mainly attributed to serum sickness and the tetanus antitoxin and were characterized as serum reactions, i.e., reactions complicated by acute carditis (3), morphologic cardiac reactions, and lesions with rheumatic carditis characteristics (4). The first publications referred to the allergic etiology of angina pectoris and myocardial infarction (5) and attempted to elucidate the clinical aspects and not the underlying pathophysiology (6). However, a comprehensive description of the allergic angina syndrome as a coronary spasm representing a manifestation of endothelial dysfunction or microvascular angina leading to allergic acute myocardial infarction has not been described since 1991 (7).

Currently, allergic angina and allergic myocardial infarction constitute coronary artery disorders that might be caused by numerous and continuously increasing causes, accompanied with broadening clinical symptoms and signs, via multi-organ arterial system involvement in patients of any age and consisting a wide spectrum of mast cell-associated disorders (2,8,9,10,11,12,13,14,15).

In this review, we present the pathophysiologic considerations of allergic angina, allergic myocardial infarction, and allergic stent thrombosis, which, together with their associations and clinical implications, constitute the manifestations of Kounis syndrome.

## Definition

**Figure 1:**
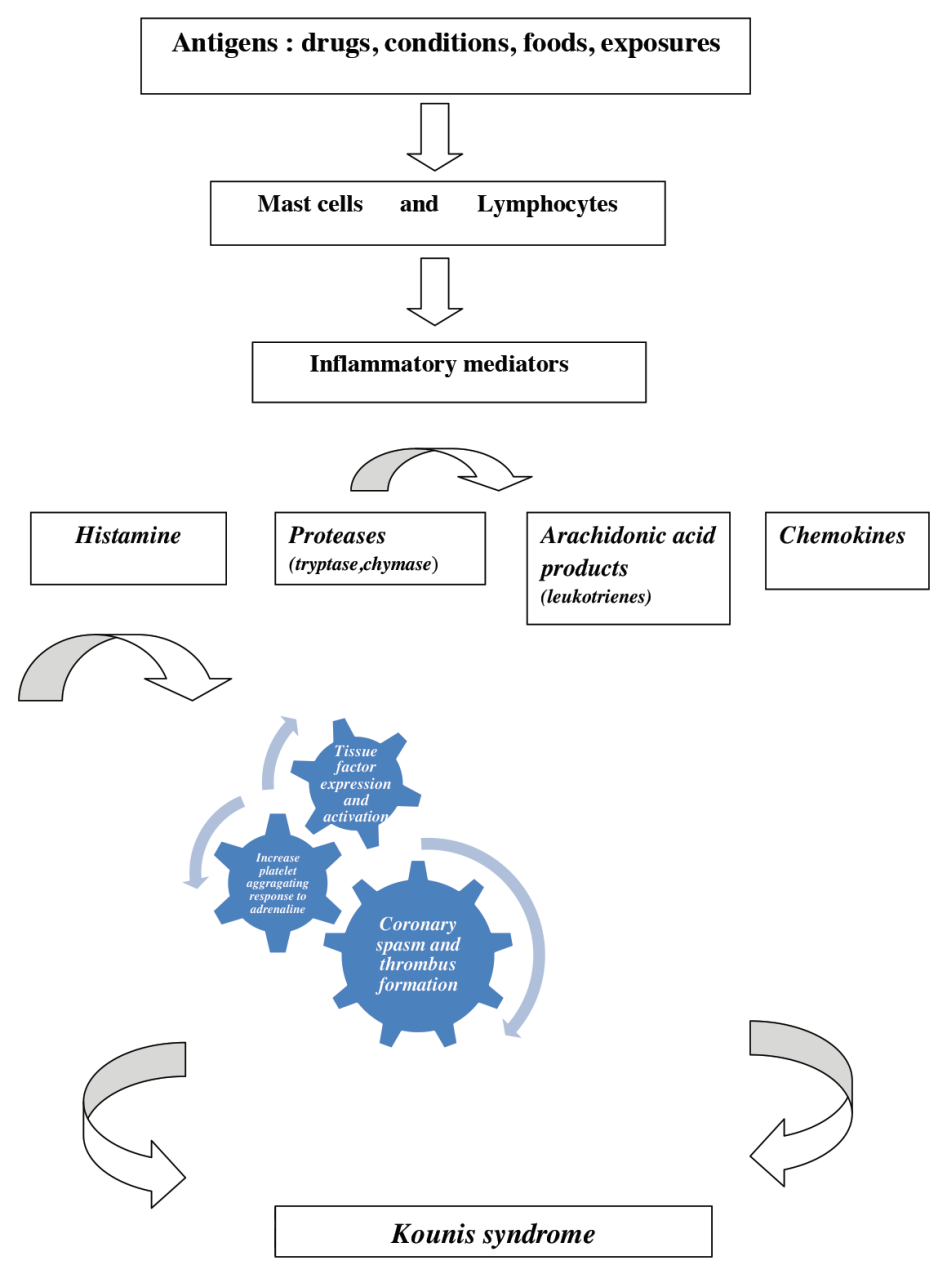
The pathophysiology of Kounis syndrome.

Coronary syndromes related to mast cell-associated disorders and inflammatory cell interactions, such as those involving T-lymphocytes and macrophages, further inducing allergic, hypersensitivity, anaphylactic, or anaphylactoid insults, are currently referred to as Kounis syndrome (Figure 1).

This syndrome is caused by inflammatory mediators released during an allergic insult, post-inflammatory cell activation, and interactions via multidirectional stimuli acting as a “ball of thread” (16). A platelet subset of 20% with high- and low-affinity IgE surface receptors is also involved in this process (17).

Kounis syndrome is not only a single-organ disease but also a complex multisystem disease; it is accompanied by allergy–hypersensitivity–anaphylaxis involving the skin, respiratory, and vascular systems in the context of anesthesia, surgery, radiology, oncology, dermatology, or even dental and psychiatric medicine; further, it significantly influences both morbidity and mortality. Kounis syndrome resembles hypersensitivity “blow up,” affecting not only the coronaries but also the cerebral (18) and mesenteric arteries (19). Mast cells enter the circulation from the bone marrow as mononuclear cell precursors and circulate as mast cell precursors (20), expressing their surface KIT receptors (21) for stem cell factor. Stem cell factor is a major cytokine that plays an essential role in the mast cell growth, survival, differentiation, proliferation, adhesion, and storage. Mast cells can penetrate all human tissues, including brain tissue, in which no allergic reactions occur owing to IgE antibodies being unable to cross the blood–brain barrier. Mast cell differentiation, growth, and maturation in all these tissues may occur over several days or even weeks. In contrast, basophils develop in the bone marrow from granulocyte precursors, enter the circulation as mature cells, and penetrate different tissues only during the later stages of an allergic reaction.

## History

Approximately 4500 years ago, i.e., in the year 2600 BC, Pharaoh Menes died suddenly while traveling to the British Isles (22); his death was attributed to a wasp or hornet sting. This event is based on the hieroglyphs of two, although only partially preserved but almost identical, ebony plates found at one of the many putative Menes burial sites (23). Mene’s death is considered the first anaphylactic death in humans.

In the modern world, the correlation between cardiovascular disorders and anaphylactic reactions was established in the previous century (24). In particular, animal experimental models (25) revealed electrocardiographic changes in dogs and rabbits but not in guinea pigs, because the later usually died due to asphyxia-induced death during the experiments (26).

A century later, in post-mortem acute myocardial infarction of non-allergic etiology, a common pathway between allergic and non-allergic coronary events was established by the Finish researchers, demonstrating a much higher (200:1) degree of mast cell degranulation at plaque erosion or rupture sites compared with at adjacent areas or even more distant segments (27). Because mast cell maturation and release of their mediators may take days or weeks, the question raised in this report was that how were such high concentrations of degranulated mast cells found at the plaque erosion or rupture sites. It seems likely that the mast cells would have already been present at the erosion or rupture sites, ready to degranulate and release their contents just before the acute coronary event. This can have profound therapeutic and clinical implications in the prevention of the coronary plaques and progression of the unstable lesions via the inhibition of mast cell degranulation and further acute myocardial infarction. Indeed, drugs and natural molecules stabilizing the mast cell membrane and monoclonal antibodies protecting the mast cell surface can emerge as novel therapeutic modalities capable of preventing acute coronary and cerebrovascular events (28). This has already been achieved in experimental models where late thrombotic events were aborted by stabilizing the mast cell membrane with sodium cromoglycate and reducing the inflammation with dexamethasone (2). In the meantime, other researchers (29) classified allergic angina as a subgroup of dynamic coronary occlusion lesions where allergic reactions with mediators, such as histamine or leukotrienes, acting on the coronary vascular smooth muscle cells can induce vasospastic angina, expressing the possibility that even ordinary allergic reactions can promote plaque disruption (30).

This unique association between anaphylaxis and the heart was published in the afore-mentioned comprehensive report published in 2006 (28), characterizing these events as the “magnificent nature’s own experiment”.

Furthermore, a potential relationship between drug-eluting coronary stent thrombosis and respective hypersensitivity to coronary stent components as a potential manifestation of Kounis syndrome was published during the same year (31).

## Causality

A variety of drugs, foods, environmental exposures, and clinical conditions can induce the Kounis syndrome (Table 1). Commonly used drugs, such as aspirin, antihypertensives, and corticosteroids as well as antibiotics and nonsteroidal anti-inflammatory drugs, constitute some of the main offenders. Aspirin alone (32) or in combination with underlying nasal polyps and asthma, known as the Samter–Beer triad (33), has been incriminated as a potential trigger for Kounis syndrome (34).

Corticosteroids constitute the main treatment of allergic, cutaneous, respiratory, rheumatologic, and renal diseases as well as immunosuppression in transplant recipients. Paradoxically, corticosteroids may themselves cause allergic reactions and even anaphylaxis. In a case report, prednisolone was administered in a young patient with normal coronary arteries for the treatment of wasp sting anaphylaxis; the patient was complicated by acute myocardial infarction, resembling the Kounis syndrome (35). As shown in Figure 1, it is anticipated that any substance acting as an allergen can act as a potential cause of Kounis syndrome.

Consumption of spoiled fish can induce Kounis syndrome via anisakiasis (36) and scombroid food poisoning (37). The first is an IgE-mediated food allergy caused by the consumption of fish contaminated with the nematode *Anisakis simplex*, which secretes allergenic substances that can further cause anisakiasis and Kounis syndrome. In such cases, future abstention from eating raw or undercooked fish or seafood is always required. The later refers to fish poisoning by gram-negative bacteria containing the enzyme histidine decarboxylase, which converts the amino acid histidine (fish component) to histamine and further causes symptoms of Kounis syndrome. Scombroid food poisoning pathophysiology is not based on an IgE-mediated food allergy, and consequently, there is no need to avoid eating fish or seafood. However, refraining from spoiled fish consumption is necessary. Dental materials (8) as well as psychiatric medications (38) have been also incriminated as causative agents.

Environmental exposure to various substances can occur; examples of this are the “kiss of death,” (39) wherein when a person after peanut consumption kisses passionately the one allergic to peanuts, and “dog licking,” (40) wherein when a dog who receives antibiotics, such as penicillin, for any infection licks his penicillin-allergic master and exposes him to penicillin concentrated in the saliva.

## Variants and clinical presentation

Cardiac symptoms, including chest pain, coronary vasospasm, angina pectoris, myocardial infarction, acute cardiac failure, and sudden cardiac death associated with subclinical, clinical, acute, or chronic allergic reactions constitute the clinical manifestations of Kounis syndrome. Up to 13% of sudden cardiac deaths in adults are associated with mast cell degranulation, denoting an underlying silent allergic reaction and the Kounis syndrome (41). Kounis syndrome is not a rare disease but is easily overlooked and infrequently diagnosed. A high index of suspicion regarding this syndrome is of paramount importance. Kounis syndrome cases, although under-reported, are more often encountered in clinical practice, and it is anticipated that many more causative factors will be implicated in the future. Any natural allergen can be a potential trigger for Kounis syndrome. It has been stated that when physicians treat patients with anaphylaxis, the possibility of Kounis syndrome should be immediately excluded by examining electrocardiographic findings and measuring troponin levels (42). Indeed, various electrocardiographic changes ranging from cardiac arrhythmias of any kind to those resembling digitalis intoxication and the ST segment elevation or depression to any degree of heart block can be associated with the cardiac symptoms and signs of Kounis syndrome (2). Three variants of Kounis syndrome have been described. The type I variant, initially described (7) as a coronary artery spasm, refers to the syndrome in patients with normal or nearly normal coronary arteries and no predisposing factors for coronary artery disease; in these patients, the acute release of inflammatory mediators can induce either coronary artery spasm without an increase in cardiac enzyme and troponin levels or coronary artery spasm progressing to acute myocardial infarction with raised cardiac biomarker and troponin levels. Today, this variant constitutes one of the causes of the myocardial infarction with non-obstructive coronary arteries (MINOCA), a new clinical entity (43), and represents a manifestation of endothelial dysfunction or microvascular angina. Type II variant (9) refers to the syndrome in patients with quiescent preexisting atheromatous disease; in such patients, the acute release of inflammatory mediators can induce either coronary artery spasm with normal cardiac biomarker and troponin levels or coronary artery spasm together with plaque erosion or rupture manifesting as acute myocardial infarction. Type III variant (44) includes patients with coronary stent thrombosis (subtype a) or stent restenosis (subtype b) due to allergic inflammation (45). Indeed, stent components that contain nickel can act as allergens, inducing both stent thrombosis and restenosis (45). Both subtypes of variant III have been diagnosed in patients with stent implantation who died suddenly and in those whom histological examination of the coronary intima, media, and/or adventitia adjacent to stent deployment showed infiltration of eosinophils and/or mast cells.

## Incidence and epidemiology

As per the first nationwide epidemiological study on Kounis syndrome conducted in the United States of America, with data derived from the National Inpatient Sample databases (46), one of the largest all-payer inpatient healthcare datasets publicly accessible during the years 2007–2014, the overall prevalence of Kounis syndrome among the patients hospitalized for allergy, hypersensitivity, and anaphylactic reactions was 1.1% (unstable angina, 0.2%; ST elevation myocardial infarction, 0.2%; and non-ST elevation myocardial infarction, 0.7%); a subsequent inpatient mortality rate of 7.0% was noted. The cohort comprised 235,420 patients, 2,616 of which were diagnosed with Kounis syndrome.

In a Turkish study (47), the annual incidence of Kounis syndrome in the emergency department among all the admissions and patients with allergy was 19.4 per 100,000 and 3.4% (27 of 793), respectively. At the Numazu City Hospital emergency department (Shizuoka, Japan), the annual incidence of Kounis syndrome from 2012 to 2017 in patients with anaphylaxis was 2% (2 of 100). One of these patients survived, but the other died (48). At the Shizuoka Hospital of Juntendo University, the annual incidence of Kounis syndrome at the emergency department from 2013 to 2017 in patients with anaphylaxis was 2.2% (3 of 138). The three patients survived, but one had anoxic encephalopathy induced by cardiac arrest (49). In these two cases at the Numazu City Hospital and Shizuoka Hospital, cardiac arrest had been induced by drugs.

Southern Europe, particularly Turkey, Greece, Italy, and Spain are the areas where Kounis syndrome has mostly been encountered. Increased awareness among physicians about the existence of Kounis syndrome, climate and environmental conditions, pollen cross reactions, hymenoptera exposures, snake bites, overconsumption of medicines, or inadequacy of preventive measures are some factors that possibly contribute to this variation (2).

## Stent thrombosis and Kounis syndrome

**Figure 2:**
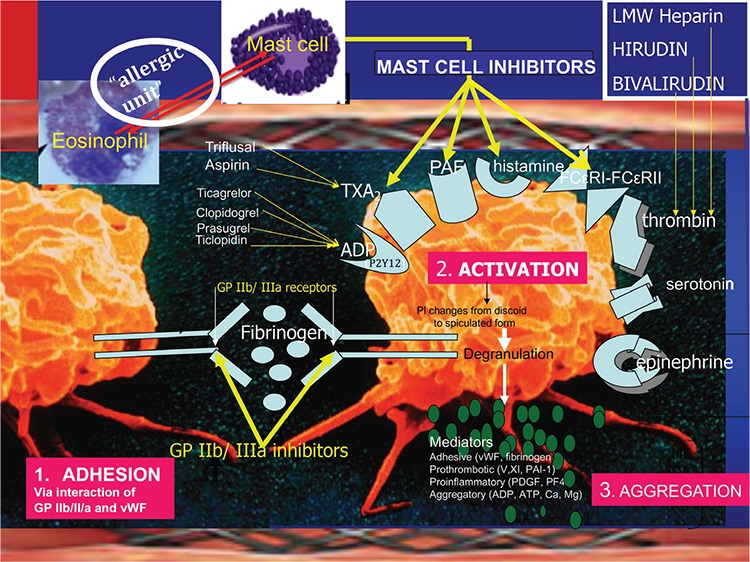
The pathophysiology of type III variant of Kounis syndrome: Platelet adhesion via interaction of glycoprotein IIb/IIIa (GP IIb/III/a) and von Willebrand factor followed by activation-aggregation leading to stent thrombosis via various platelet receptors including allergic receptors for histamine, platelet-activating factor, thromboxane, and FCεRI-FCεRII receptors. ADP: adenosine diphosphate; PAF: platelet-activating factor; TXA: thromboxane; vWF: von Willebrand factor

Currently, the most frequently performed therapeutic procedure in medicine is percutaneous transluminal coronary angioplasty with coronary stent implantation, and this procedure has become a lifesaving medical technology (50). Three types of stents are used today. The first is bare metal stents comprising a 316-L stainless steel platform containing nickel, chromium, titanium, manganese, and molybdenum (51); second, drug-eluting stents that also dispose a stainless steel platform but with cobalt or platinum alloys and a biocompatible durable or biodegradable polymer combined with the antiproliferative drug everolimus or zotarolimus (52); and third, bioresorbable stents. Stent restenosis and thrombosis with a mortality rate of up to 50% are the main complications with these devices. In all the clinical reports and pathologic findings in patients subjected to thrombus aspiration or in those who died following stent thrombosis and the animal experimental studies, the thrombus had been shown to be infiltrated by various interrelated and interacting inflammatory cells including eosinophils, macrophages, T cells, and mast cells, pointing toward a hypersensitivity inflammation (53). All stent components, namely polymer coating, stent platforms with their metals, and the released drugs can act as strong antigens that exert continuous, repetitive, persistent, and chronic hypersensitivity irritation to the coronary intima. We have defined an “antigenic complex” of polymers, nickel alloys, and eluted drugs that come in contact with the delicate coronary intima. This complex, together with the concomitant administration of oral antiplatelet drugs and environmental exposures, can induce hypersensitivity inflammation and stent thrombosis as a manifestation of Kounis syndrome (54). Hence, the type III variant of Kounis syndrome has been established. The mechanism of stent thrombosis and Kounis syndrome is depicted in Figure 2. Practicing physicians should never forget that “the more allergens an atopic patient is exposed to, the easier and quicker anaphylaxis and Kounis syndrome appear” (55).

## The ATAK complex and the Kounis syndrome

The ATAK complex (Adrenaline, Takotsubo, Anaphylaxis, and Kounis syndrome) needs “attacking” to elucidate its etiology and pathophysiology and to apply proper preventive and therapeutic measures (56). Takotsubo syndrome has several names, such as stress-induced cardiomyopathy, transient left ventricular apical ballooning, apical ballooning syndrome, atypical apical ballooning, ampulla cardiomyopathy, broken heart syndrome, or transient left ventricular dysfunction syndrome. Stress generates impulses from high cortical centers that, via the limbic system of the hypothalamus, activate subcortical structures and release neurohormones, such as epinephrine and cortisol, modulating acute-phase responses by cytokine production and further affecting the cardiovascular system (57).

Several reports have correlated Takotsubo cardiomyopathy with Kounis syndrome (58). Takotsubo cardiomyopathy has been observed not only in patients suffering from anaphylaxis but also in those simply observing and assisting the treatment of anaphylaxis. This confirms that Takotsubo cardiomyopathy represents stress-induced myocardial stunning.

Therefore, a vicious cycle that makes the ATAK complex difficult to explain exists. Because anaphylactic reactions can also induce Takotsubo syndrome, we have proposed the measurement of anaphylactic inflammatory mediators such as histamine, tryptase, chymase, leukotrienes, thromboxane, and platelet-activating factors as well as the use of corticosteroids or mast cell stabilizers for the prevention of this condition (59). Differentiation between Takotsubo cardiomyopathy and Kounis syndrome can be difficult, and the treatment of suspected Kounis syndrome with epinephrine may precipitate or exacerbate Takotsubo cardiomyopathy (60).

## Kounis syndrome as a predominant feature of anaphylaxis

Clinical and laboratory evidence have indicated that the heart, particularly the coronary arteries, constitute the primary targets of allergic inflammatory mediators where Kounis syndrome is the predominant feature.

Independent of the reaction severity, cardiac dysfunction may develop during anaphylactic reactions and may resolve with suitable treatment post-anaphylaxis resolution (61). Using the QLAB-CMQ software program of the Speckle tracking echocardiography and by measuring global longitudinal strain via apical chambers (4, 3, and 2 chambers) through at least four consecutive cardiac cycles, the global longitudinal strain value has been demonstrated to be lower shortly after anaphylaxis than at 6 weeks later. This finding was evident in 50% of the patients, whereas global longitudinal strain was reportedly much lower in the anaphylaxis group than in the equivalent group with urticaria. Indeed, a lower global longitudinal strain has been recently associated with a higher long-term risk of cardiovascular morbidity and mortality in a low-risk general population (62,63).

As per another report, cardiac troponin I levels were significantly increased in 31 patients admitted to the emergency department due to anaphylaxis, angioedema, urticaria, and urticaria angioedema, compared with 125 healthy controls (64). The cardiac troponin I levels were higher in patients with anaphylaxis than in those with milder allergic reactions. These findings might have profound clinical, therapeutic, and pathophysiologic implications as far as anaphylaxis, myocardial injury, and Kounis syndrome are concerned.

Furthermore, several animal studies have also shown that the heart, particularly the coronary arteries, could serve as a primary substrate in experimental anaphylaxis.

Moreover, decrease in cardiac output by 90%, acute myocardial ischemia in the electrocardiogram, an initial increase in the arterial blood pressure by 35% followed by a decreace after 4 min, and a significant increase in the left ventricular end-diastolic pressure by 35% indicating pump failure had been observed in challenged ovalbumin-sensitized guinea pigs. The conclusion was that the anaphylactic cardiac damage might have been caused by peripheral vasodilatation and that the volume loss due to an increase in vascular permeability could definitely be excluded (65).

However, anaphylaxis in sensitized mice induced an initial transient increase followed by a progressive decrease in post-antigen injection in aortic blood flow and mean arterial pressure, which were attenuated by pretreatment with either a platelet-activating factor receptor antagonist or the histamine H1 receptor antagonist diphenhydramine and were further abolished by their combination. Furthermore, no total peripheral resistance reduction was observed. Thus, the authors again concluded that mice anaphylactic hypotension could be only attributable to cardiac output reduction via platelet-activating factor and histamine actions and not to vasodilation (66).

## Anesthesia and Kounis syndrome

Diagnosing anaphylaxis in anesthesia is challenging because cutaneous manifestations, such as flushing, urticaria, and angioedema, may be absent. Several causes that can induce anaphylactic reactions and Kounis syndrome are encountered during anesthesia. Neuromuscular blocking drugs, antibiotics, latex exposure, contrast media, hypnotic agents, opioids, colloids, apronitin, protamine, chlorhexidine, dyes, local anesthetics, blood transfusion, and passive transfer of peanut allergens in blood products are some of the offenders. Anaphylaxis may occur at any time during anesthesia and with all potentially allergenic substances; however, it usually occurs shortly after induction.

Several grading systems have been introduced for the classification of anaphylaxis during anesthesia. According to the Ring and Messmer system, which is the most frequently used (67), anaphylactic symptoms and signs are graded as follows: grade I, which involves the cutaneous–mucus signs; grade II, which involves mild cutaneous–mucus signs that may be combined with cardiorespiratory signs; grade III, which involves cutaneous–mucus signs and/or bronchospasm with cardiovascular collapse; and grade IV, which denotes cardiac arrest. The grades ΙΙΙ and IV may correlate with Kounis syndrome symptomatology (68,69). Thus far, several conditions, such as mastocytosis, left parotid gland excision, spinal anesthesia, laparoscopic ileocecal excision for cecal cancer, Takotsubo syndrome, coronary artery bypass graft, have been associated with the occurrence of Kounis syndrome during anesthesia and with the variety of drugs used for anesthesia.

## Surgery and Kounis syndrome

During surgery, several substances such as drugs, metal devices, polymers, latex, disinfectants, or infusion materials are used and may act as antigens able to induce hypersensitivity or anaphylactic reactions and Kounis syndrome. Extremely useful therapeutic inventions such as artificial cardiac valves, pacemakers, defibrillators, coronary stents, left ventricular assist devices, aortic grafts, stents for femoral arteries or metal prostheses used in orthopedics, and neurosurgery can induce foreign body reactions and hypersensitivity reactions due to their metal, polymer, and eluting drug ingredients. Several reports have correlated Kounis syndrome with device implantation. For example, Kounis syndrome with cardiogenic shock occurred during transfemoral transcatheter aortic valve replacement (70). The SAPIEN bovine valve (Edwards Lifesciences, Irvine, California) has a polyethylene terephthalate skirt and a cobalt chromium stent. Both CoreValve porcine pericardium valve (Medtronic, Minneapolis, Minnesota) and Portico bovine pericardium valve (St. Jude Medical, Minneapolis, Minnesota) consist of a nitinol (nickel-titanium alloy) stent. Metal anions can induce Kounis hypersensitivity-associated thrombotic syndrome via platelet FCγRI, FCγRII, FCεRI, and FCεRII receptors (16,17). Specifically, there had been a case of female patient who developed repeated cardiac arrests and died during acromioclavicular resection and partial supraspinatus tear and labrum tear debridement via arthroscopy. The Kounis syndrome via the ATAK complex was incriminated (58). Thus, we recommend mandatory history reviewing and scrutinizing, for any drug reaction before surgery or any device implantation. Even the most inert materials such as titanium have been incriminated as able to induce hypersensitivity reactions and Kounis syndrome by bio-corrosion (71).

## Radiology and Kounis syndrome

Contrast media that are used routinely in radiology have also been associated with Kounis syndrome.

Iodine-based contrast media, used in radiology, are usually classified as ionic or nonionic. The low-osmolarity non-anionic contrast materials demonstrate lower side effects, at approximately 2.1%, compared to the anionic ones. However, mild symptoms that include fever, purpura, skin reactions, convulsions, dyspnea, renal damage, and the severe reactions such as hypersensitivity reactions including Kounis syndrome (72) and death (73) can be encountered during their use. Nonionic and dimeric contrast molecules bind to circulating proteins and create allergenic haptens to a lesser extent than the ionic and monomeric contrast materials. Several conditions and co-existing medications have been identified as risk factors for the occurrence of side effects of iodinated contrast materials.

Gadolinium is silvery-white metal and gadolinium-based contrast agents are used to improve the visibility of internal body structures in magnetic resonance imaging. This type of contrast agents may also be associated with the development of Kounis syndrome (74), however, paradoxically, are currently used for the Kounis syndrome diagnosis! (75).

## Oncology and Kounis syndrome

Cancer and cardiovascular disease constitute the two major causes of death worldwide, along with chemotherapy, radiation therapy, and surgery, the main therapeutic modalities. Chemotherapy can induce cardiovascular deterioration manifesting acute and chronic symptoms, commonly referred to as cardiac toxicity. Cardiac toxicity might be acute, sub-chronic, and chronic and is a dose-dependent cardiovascular adverse reaction that persists despite the discontinuation of the causative treatment. The outcome of cardiac toxicity is a fibrotic response that should be confirmed histologically, a procedure that has not been discussed up to date. The final pathophysiological manifestation is global or septal cardiomyopathy with reduced left ventricular ejection fraction.

However, most chemotherapeutic agents can induce hypersensitivity reactions. Cardiac hypersensitivity refers to an inflammatory response that is not dose-dependent, that may arise at any time during treatment, even with minimal drug concentrations, and is accompanied by anti-drug antibodies of IgG and IgE type. Indeed, chimeric monoclonal antibodies (named after the Greek mythological monstrous hybrid chimera, composed of parts of more than one animal such a lion, with the head of a goat fire-breathing, a tail end with snake's head, and which is one of the offspring of Typhon and Echidna and a sibling of monsters Cerberus and the Lernaean Hydra) such as anti-TNF-α, non-TNF blocking, and the epidermal growth factor receptor inhibitors have induced hypersensitivity reactions including Kounis syndrome up to 25.7%, platinum agents up to 27%, and taxanes up to 30% (76,77).

## Dermatology and Kounis syndrome

The human skin constitutes the target of the inflammatory mediators released from the mast cells and eosinophils during an allergic or anaphylactic episode. The allergic skin manifestations can be immediate, delayed, or absent. Urticaria, rash, erythema, and angioedema are some examples accompanying the Kounis syndrome and helping in its diagnosis. Delayed skin manifestations are thought to be due to vasoconstriction, induced by reduced cardiac output and hypotension during rapidly progressive anaphylaxis. The absence of skin manifestations (78) has been attributed to shock, due to reduced cardiac output because of leakage of plasma and volume loss that reduces the venous return and prevents or delays the released anaphylactic mediators to reach and exert their action in skin areas, and induce urticaria, rash, or erythema (14). On the other hand, chronic urticaria itself can be the cause of Kounis syndrome! (79).

## Diagnosis

Patients with systemic allergic reactions associated with clinical, electrocardiographic, angiographic echocardiographic, and laboratory findings of acute myocardial ischemia should be diagnosed as having Kounis syndrome. Serum tryptase, histamine, immunoglobulins (IgE), cardiac enzymes, and cardiac troponins are helpful to confirm the diagnosis. Tryptase should be measured half an hour after the initial symptoms and every 30 min thereafter during the following 2 h (80). In case of death, aortic postmortem tryptase measurements can be proven valuable. Histamine is short living and circulates for only 8 min after an allergic event; therefore, blood samples should be collected immediately after the onset of chest pain and before any analgesic, especially morphine, administration.

In Kounis syndrome, the newer techniques such as thallium-201 single-photon emission computer tomography (SPECT) and 125I-15-(p-iodophenyl)-3-(R,S) methyl pentadecanoic acid (BMIPP) SPECT have revealed severe myocardial ischemia, while coronary angiography showed normal coronary arteries (81). Furthermore, the delayed contrast-enhanced images of cardiac magnetic resonance imaging have shown normal washout in the subendocardial lesion area in patients with Kounis syndrome, type I variant (75).

## Treatment

Kounis syndrome should be treated with careful attention, because the drugs administered to treat cardiac manifestations can worsen allergy, and the drugs given for the allergic symptoms can aggravate the cardiac dysfunction.

In type I variant, treatment includes intravenous corticosteroids such as hydrocortisone at a dose of 5 mg/kg/day and H1 and H2 antihistamines such as diphenhydramine at a dose of 1-2 mg/kg and ranitidine at a dose of 1 mg/kg. Vasodilators such as calcium channel blockers and nitrates can abolish the vasospasm. Intravenous or sublingual nitroglycerin seems reasonable and safe in patients with Kounis syndrome, if the blood pressure is satisfactory. Most patients have demonstrated well tolerance to oral and sublingual nitroglycerin (2). Bolus administration of antihistamines should be performed slowly, as these drugs can precipitate hypotension and compromise the coronary flow.

In type II variant, treatment includes an acute coronary event protocol together with the corticosteroids and antihistamines. Vasodilators such as nitrates and calcium channel blockers are given when they are necessary. Beta-blockers can exaggerate coronary spasm due to unopposed action of alpha-adrenergic receptors. Epinephrine can aggravate ischemia and worsen coronary vasospasm. Thus, sulfite free epinephrine is preferable to be given intramuscularly at doses 0.2-0.5 mg (1:1000). Moreover, an aqueous solution is preferable. Epinephrine may be ineffective in patients already on beta-blockers.

Glucagon infusion (1-5 mg, intravenously over 5 min, followed by infusion 5-15 μg/min) can be given in this case (82). Methoxamine, a potent alpha agonist, can also be considered in patients who do not respond to epinephrine (82). Opiates such as morphine, codeine, and meperidine should be administered with extreme caution, since they can induce massive mast cell degranulation and aggravate the allergic reaction (83). Fentanyl and its derivatives show slight mast cell activation and are preferable. Paracetamol (acetaminophen) is not recommended, especially its intravenous administration, because it might cause severe hypotension due to reduction of cardiac output (84).

In type III variant, treatment includes the current acute myocardial infarction protocol, urgent aspiration of intrastent thrombus, and its histological examination with staining for eosinophils (hematoxylin and eosin) and mast cells (Giemsa). Allergic symptoms and signs following stent implantation need antihistamines, corticosteroids, and mast cell stabilizers. When symptoms persist, identification of the culprit with patch and/or prick skin tests and desensitization strategies should be applied. If these measures fail, stent extraction seems unavoidable (85).

Kounis syndrome is a unique complex cause of acute coronary syndrome that requires proper diagnosis, immediate decisions, and rapid treatment. A high index of suspicion, when dealing with an allergic reaction, is of paramount importance. It needs a full cardiologic work-up, including a 12-lead electrocardiogram, echocardiogram, angiogram, and cardiac risk factor modification, following the acute phase. An atopic work-up should be performed including the assessment of any food allergies, insect stings, Hymenoptera bites, drugs, and other environmental agents or conditions. Skin patch or prick tests and food challenges may be additional useful measures for the culprit cause identification.

## Figures and Tables

**Table 1:**
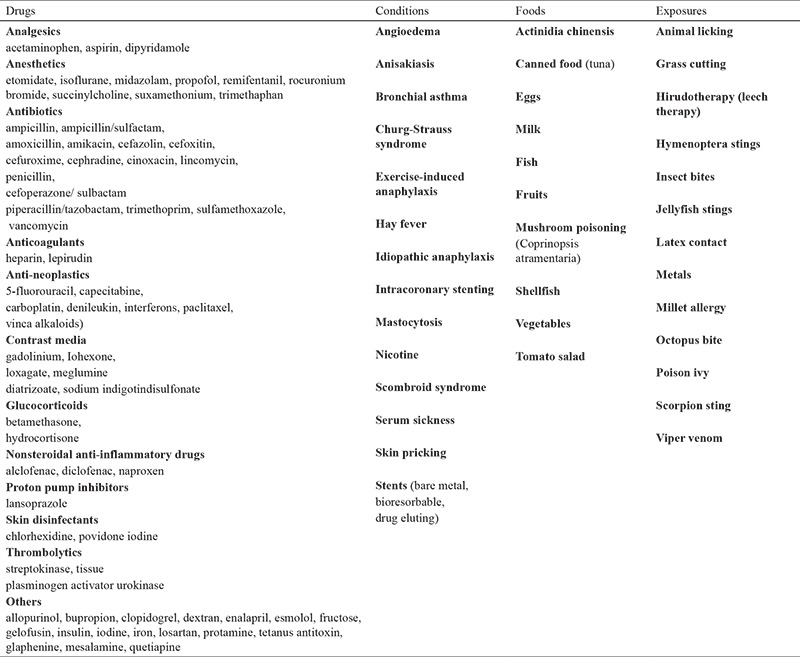
Reported causes of Kounis syndrome
